# Efficacy and safety of novel carbapenem-β-lactamase inhibitor combinations: imipenem-cilastatin/relebactam results from randomized controlled trials

**DOI:** 10.3389/fmed.2023.1304369

**Published:** 2023-12-21

**Authors:** Qingxin Yang, Yanqiu Yang, Rong He, Bin Yu, Yueling Zhong, Fei Lin

**Affiliations:** ^1^Pharmaceutical Department, Mianyang Orthopaedic Hospital, Mianyang, China; ^2^Department of Science and Technology, The First Affiliated Hospital of Chengdu Medical College, Clinical Medical College, Chengdu Medical College, Chengdu, China; ^3^Department of Respiratory and Critical Care Medicine, The First Affiliated Hospital of Chengdu Medical College, Clinical Medical College, Chengdu Medical College, Chengdu, China; ^4^Department of Pharmacy, Mianyang Central Hospital, Mianyang, China; ^5^Department of Pharmacy, The First Affiliated Hospital of Chengdu Medical College, Clinical Medical College, Chengdu Medical College, Chengdu, China

**Keywords:** imipenem/cilastatin/relebactam, novel carbapenem-β-lactamase inhibitor combination, efficacy, safety, bacterial infections

## Abstract

**Background:**

Gram-negative bacteria is a global public health problem. Treatment options include novel beta-lactamase inhibitors.

**Objectives:**

The objective of this study was to collect information on the efficacy and safety of novel β-lactamase inhibitor combinations such as imipenem-cilastatin/relebactam (IMI/REL).

**Methods:**

In order to comprehensively evaluate the clinical, microbiological, and adverse events outcomes, a meta-analysis was conducted on clinical trials comparing novel β-lactamase inhibitor combinations with existing comparator therapies.

**Results:**

Four studies comprising 948 patients were included in the analysis. IMI/REL therapy demonstrated similar clinical responses to comparators across various treatment visits, including discontinuation of intravenously administered therapy visits [DCIV, RR = 1.00 (0.88, 1.12)], early follow-up visits [EFU, RR = 1.00 (0.89, 1.14)], late follow-up visits [LFU, RR = 1.00 (0.88, 1.13)]. Moreover, no significant difference in the microbiologic response of MITT patients was observed between IMI/REL and comparators across DCIV [RR = 0.99 (0.89, 1.11)], EFU [RR = 1.01 (0.95, 1.07)], and LFU visits [RR = 1.00 (90.94, 1.07)]. In terms of safety, therapy with IMI/REL and comparators exhibited similar risks of at least one adverse event (AE), drug-related AEs, and discontinuation due to AEs. The incidence of serious AEs (SAEs) was significantly lower in the IMI/REL group compared to the comparison groups. The predominant AEs were gastrointestinal disorders, with no significant difference observed between the IMI/REL group and comparators.

**Conclusion:**

The clinical and microbiologic response to IMI/REL in the treatment of bacterial infection was comparable to that of the comparator. Furthermore, the incidence of AEs and the tolerability of IMI/REL were similar among the comparators. Based on these findings, IMI/REL can be considered as a viable alternative treatment option.

## Introduction

In recent years, the misuse and overuse of antibiotics has contributed to a troubling increase in bacterial resistance. This rise in bacterial resistance is a significant global health concern. It is projected that by the year 2050, multidrug resistance will lead to 10 million deaths and result in economic losses of $100 trillion globally (1). Among the bacteria that has become resistant to antibiotics are *ESKAPE pathogen (Enterococcus faecium*, *Staphylococcus aureus*, *Klebsiella pneumoniae*, *Acinetobacter baumannii*, *Pseudomonas aeruginosa*, and *Enterobacter species). This group of antibiotic-resistant bacteria is commonly referred to as ESKAPE* (2, 3). Among these drug-resistant bacteria, gram-resistant bacteria pose a particularly significant challenge worldwide. In the World Health Organization’s (WHO) report on the pathogens that require research and development of new antibiotics, priority was given to carbapenem-resistant *A. baumannii* (CRAB), carbapenem-resistant *P. aeruginosa* (CRPA), and carbapenem-resistant *Enterobacteriaceae* (CRE) (3). It is crucial to urgently address and control the spread of infections caused by carbapenem-resistant gram-negative bacteria in hospital settings.

Imipenem/cilastatin/relebactam (IMI/REL) is a newly developed carbapenem-β-lactamase inhibitor combination marketed under the brand name “RECARBRIO” (4, 5). It consists of three active ingredients: imipenem (500 mg), cilastatin (500 mg), and relebactam (250 mg), with a total weight of 1.25 g. Relebactam (REL) exhibits broad-spectrum β-lactamase activity. It effectively inhibits both Class A (including ultra-broad-spectrum β-lactamases and KPC) and Class C (AmpC) enzymes. By protecting imipenem from degradation by specific serine β-lactamases, REL targets imipenem-resistant gram-negative bacterial strains. Consequently, the addition of relebactam enhances the sensitivity of these strains to imipenem. IMI/REL has been approved as an anti-infective agent for the treatment of specific infections caused by gram-negative bacteria that are susceptible to it. This approval is applicable to patients who are 18 years of age or older and lack alternative treatment options or have limited options available to them (6). The approved indications include hospital-acquired bacterial pneumonia (HABP), ventilator-associated bacterial pneumonia (VABP), complicated urinary tract infections (cUTIs), including pyelonephritis, and complicated intra-abdominal infections (cIAIs). IMI/REL has demonstrated effectiveness *in vitro* against a broad range of pathogens, including multidrug-resistant (MDR) *P. aeruginosa,* CRE, CRAB, and metallo-lactamase-producing *Enterobacteriaceae* (7–9). This makes it a valuable treatment option for infections caused by these challenging and antibiotic-resistant pathogens. The administration of IMI/REL is via intravenous (IV) infusion, given over a period of 30 min, every 6 h to patients 18 years of age and older with a creatinine clearance of 90 mL/min or greater. It is important to consider the patient’s renal function when dosing IMI/REL to ensure appropriate and safe administration. It is worth noting that healthcare professionals should refer to the prescribing information and consult with a pharmacist or infectious disease specialist for precise dosing recommendations and guidelines related to IMI/REL usage.

As time progresses, there have been differing findings from various studies regarding the clinical efficacy and safety of IMI/REL when compared to other antibiotics (8, 10). Some studies suggest that IMI/REL exhibits superior clinical efficacy and safety, while others report no significant difference in efficacy but a higher incidence of certain adverse events. Given the increasing prevalence of drug-resistant bacteria and the expanding indications for the use of IMI/REL in bacterial infections, it is crucial to conduct a systematic evaluation of its clinical efficacy and safety. The objective of this study was to compare the clinical efficacy and safety of IMI/REL, a novel combination of a well-established β-lactam and a novel β-lactamase inhibitor antibacterial drug, in the treatment of bacterial infections. The intent was to provide a reference for its clinical application.

## Methods

### Protocol

The Population, Intervention, Comparison, and Outcome (PICO) model was established to extract pertinent information from each study. Table 1 shows a summary of the data that will be extracted and used in relation to the review question. Our search focused on clinical trials that looked at the effects of IMI/REL on patients with bacterial infections. The goal of this research was to identify clinical trials that looked into the effects of IMI/REL on patients with bacterial infections. All the patients requiring hospitalization and intravenous antibacterial therapy that were clinically suspected and/or bacteriologically documented in this study. The following criteria were used to include participants in this study: randomized controlled trials (RCTs); patients with bacterial infection; treatment with IMI/REL or another antibacterial drug or a placebo; and efficacy and safety as outcomes. The primary outcome of this study was the clinical response and microbiologic response, which were assessed centrally and defined differently for each type of infection based on regulatory guidance. Clinical response was defined as the resolution of baseline signs and symptoms, while microbiologic response was defined as the eradication of baseline pathogens. Patients who either died or had missing data were considered treatment failures. The following study has been excluded from the PICO model: (1) *in vitro* study; (2) animal study; (3) pharmacokinetic and pharmacodynamic studies; (4) studies with no comparison group; (5) non-randomized trials; (6) healthy volunteers and animals; (7) case reports; (8) non-English literature; (9) studies lacking literature data; (10) system evaluation, or abstract.

**Table 1:** PICO Model in the study.

Population	Patients diagnosed with bacterial infection disease
Intervention	Imipenem/cilastatin/relebactam
Comparisons	Others antibacterial drug or placebo
Outcome measures	Efficacy and safety

### Data sources and selection criteria

The meta-analysis study was performed according to the PRISMA guideline (11). A systematic search was performed using the medical subject headings (“imipenem, cilastatin, and relebactam”) and entry terms in the PubMed, Embase, and Cochrane Library databases from inception to October 4, 2022, and the language was restricted to English. After finishing the research, the Endnote X 8 software (Thomson Research, United States) was used to analyze and remove the duplicate literature records. Two researchers independently read the literature title and abstract of the records for preliminary screening and read the full text of the literature likely to meet the inclusion criteria (QXY and YQY). Any disagreement over the included literature should be resolved through negotiation, discussion, or the intervention of a third reviewer (BY).

### Data collection and quality assessment

The data included the basic characteristics of the study, the intervention measures, the background treatment, the course of treatment, the outcome index, the authorship, the year of publication, the study design, the study duration, the study site, the study population, the clinical response and microbiologic response, and the risk of adverse events (AEs). Two investigators (RH and YLZ) independently extracted all data from the included studies on the following: the background treatment, the course of treatment, the outcome index, the authorship, the year of publication, the study design, the study duration, the study site, the study population, the clinical response and microbiologic response, and the risk of AEs. The different opinions on the same document should be resolved through negotiation, discussion, or intervention by the third reviewer (FL). The study did not require ethical approval.

The risk of bias for each included study was assessed using the Cochrane Collaboration’s bias assessment tool and a summary of the findings was provided (12). The tool was used by two researchers to subjectively assess the risk bias of the included studies (13). The risk of bias for each study was evaluated using the Cochrane Collaboration’s bias assessment tool, categorizing judgment as “high risk,” “low risk,” or “unclear” based on the following criteria: Random sequence generation; Allocation concealment; Blinding of participants and personnel; Blinding of outcome assessment; Incomplete outcome data; Selective reporting; and Other biases. Additionally, the quality of the evidence was assessed using the Grading of Recommendations, Assessment, Development, and Evaluation (GRADE) approach (14).

### Statistical analysis

The statistical analyses for this meta-analysis were conducted using Review Manager version 5.4. Risk ratios (RRs) and corresponding 95% confidence intervals (CIs) were used as measures of association between the outcomes and the use of IMI/REL. The heterogeneity of the included studies was assessed using the Chi-squared-based Cochran’s Q statistic and I^2^. A significance level of *p* > 0.10 or I^2^ > 50% was considered indicative of significant heterogeneity. In cases of homogeneity, a fixed-effect model was applied, whereas a random-effect model was utilized for significantly heterogeneous data. To assess the robustness of the findings, a sensitivity analysis was conducted using a leave-one-out approach, sequentially excluding each study and reassessing the results. A significance level of *p* > 0.05 was considered indicative of no significant difference.

## Results

### PRISMA summary of results

**Figure 1:**
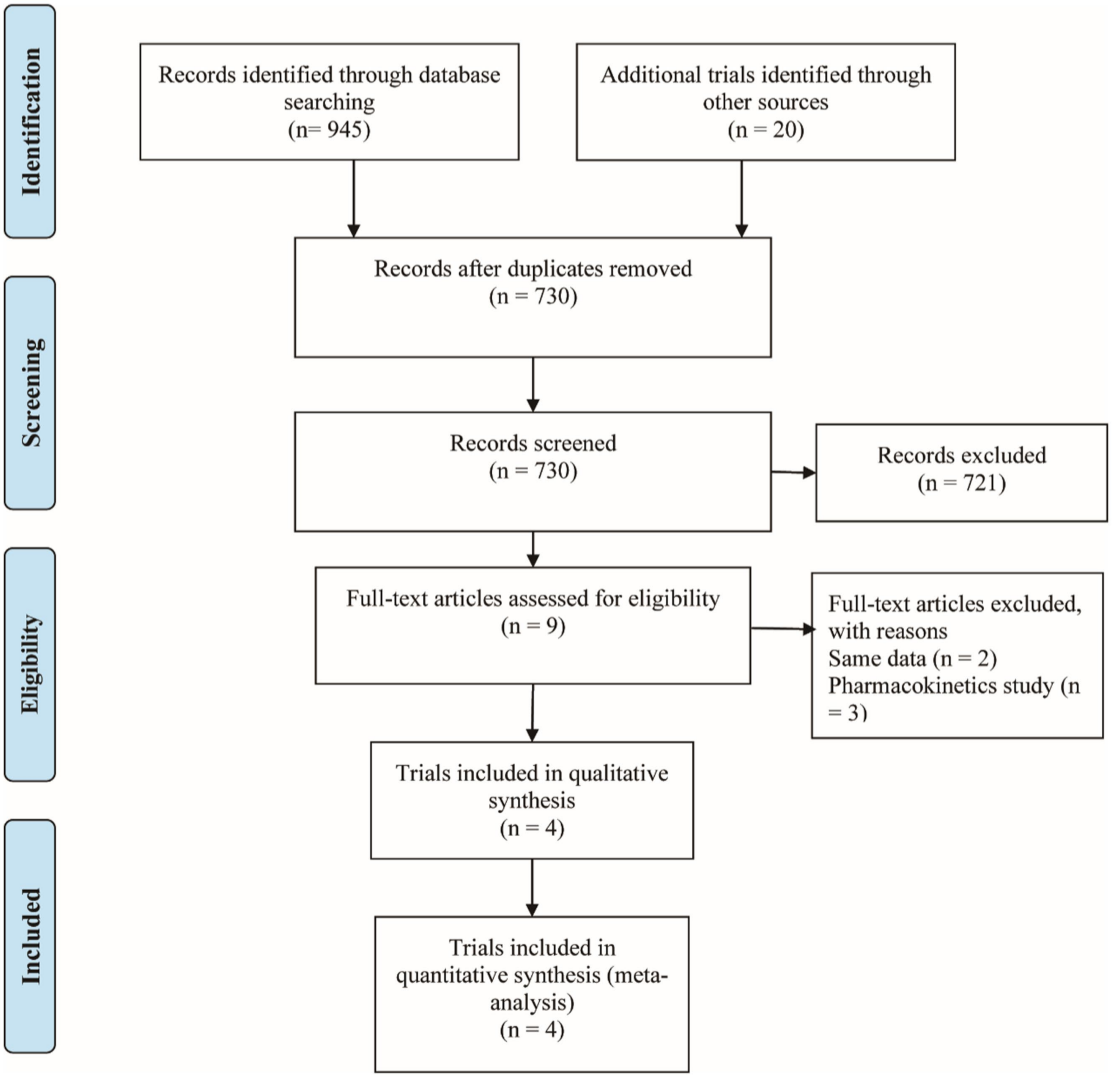
The Relebactam flow diagram.

**Figure 2:**
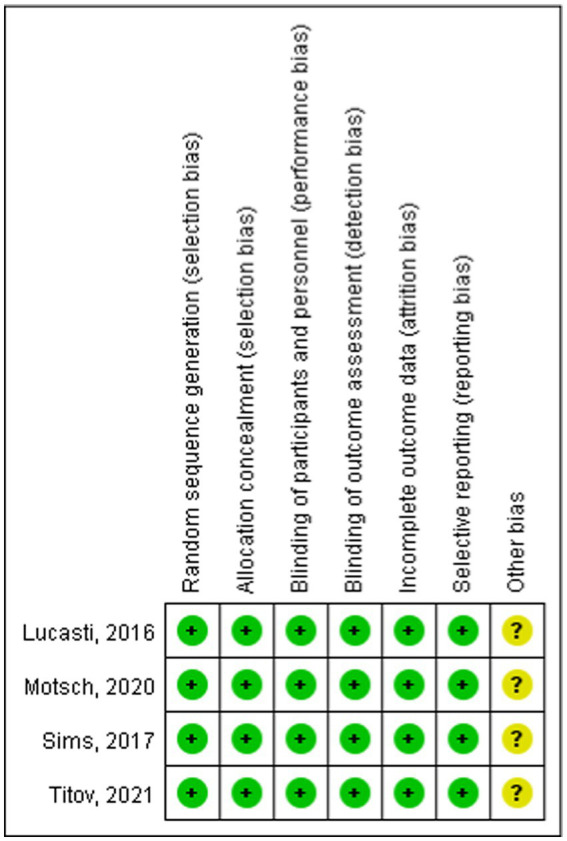
Risk of bias summary.

**Figure 3:**
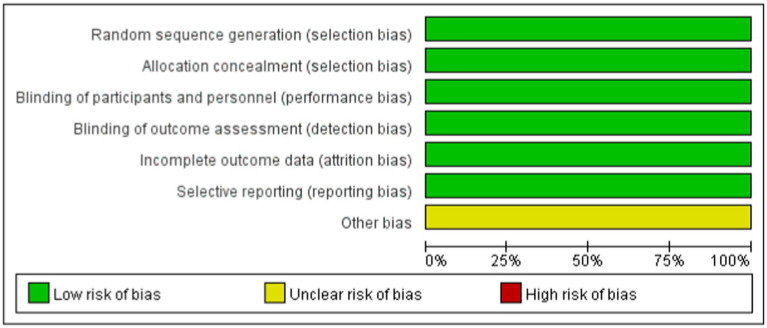
Risk of bias graph.

Figure 1 shows the PRISMA results from the database search, where a total of 965 references was found from four different databases, which included data from PubMed (N = 287), Embase (N = 611), Cochrane Library (N = 47), and Clinical Trials (N = 20). After excluding 235 duplications, the remaining 730 abstracts were further screened. Assessment of nine retrieved full-text articles excluded five studies, leaving four studies. In total, 4 studies with 948 patients were finally included in this study. Three articles were published from 2016 to 2021 (Table 2) (15–18). The Lucasti study and Sims study only included the 250 mg REL plus IMI group as the recommended dosage of the IMI/REL label. In this meta-analysis, the comparator drug in four studies is IMI plus placebo, colistin with IMI, and piperacillin/tazobactam. The risk of bias in the included studies was presented in Figures 2, 3. All of the studies were free of bias. Quality assessment using GRADE criteria collected high-quality evidence from all analyses conducted due to large numbers of participants and blindness in most studies.

**Table 2:** Characteristics of four studies in the study.

Study, year published	Study design	Study duration	Study site	Study population	No. of patients (ITT population)		Therapy Duration
Sims et al. (2017) NCT01505634 (MK-7655-003) (15)	randomized, double-blind, multicenter, Phase 2b	December 2012 to July 2015.	34 hospitals in 11 countries	18 years old with either clinically suspected and/or bacteriologically documented cUTIs or acute pyelonephritis, requiring hospitalization and iv antibacterial therapy.	IMI/REL 250 mg (*N* = 71)	IMI 2 g (*N* = 80)	5–14 days
Lucasti et al. (2016) NCT01506271 (MK-7655-004) (16)	multicenter, double-blind, randomized	November 2012 to August 201	45 sites in 20 countries	18 years of age with clinically suspected and/or bacteriologically documented cIAI requiring hospitalization and treatment with intravenous antibiotic therapy.	IMI/REL 250 mg (*N* = 118)	IMI 2 g (N = 117)	4–7 days, maximum 14 days
Motsch et al. (2020) NCT02452047(RESTORE-IMI 1) (17)	phase 3, randomized, double-blind	October 2015 and September 2017	16 sites in 11 countries	18 years old, hospitalized, and required intravenous antibacterial treatment for HAP/ VAP, cUTIs, or cIAIs	IMI/REL 250 mg (*N* = 21)	Colistin (150 mg q12h) + IMI (500 mg) (N = 10)	5–7 days, maximum 21 days
Titov et al. (2021) NCT02493764 (RESTORE-IMI 2) (18)	phase 3, randomized, double-blind	January 2016 and April 2019	113 hospitals in 27 countries	18 years old and required intravenous antibacterial therapy for nonventilated HABP, ventilated HABP, or VABP.	IMI/REL 250 mg (*N* = 264)	PIP/TAZ 4 g/500 mg (N = 267)	7–14 days

HAP: hospital acquired pneumonia; VAP: ventilator-associated pneumonia; cUTIs: complicated urinary tract infections; cIAIs: complicated intraabdominal infections; IMI/REL: imipenem/cilastatin/relebactam.

### Clinical and microbiologic response

**Figure 4:**
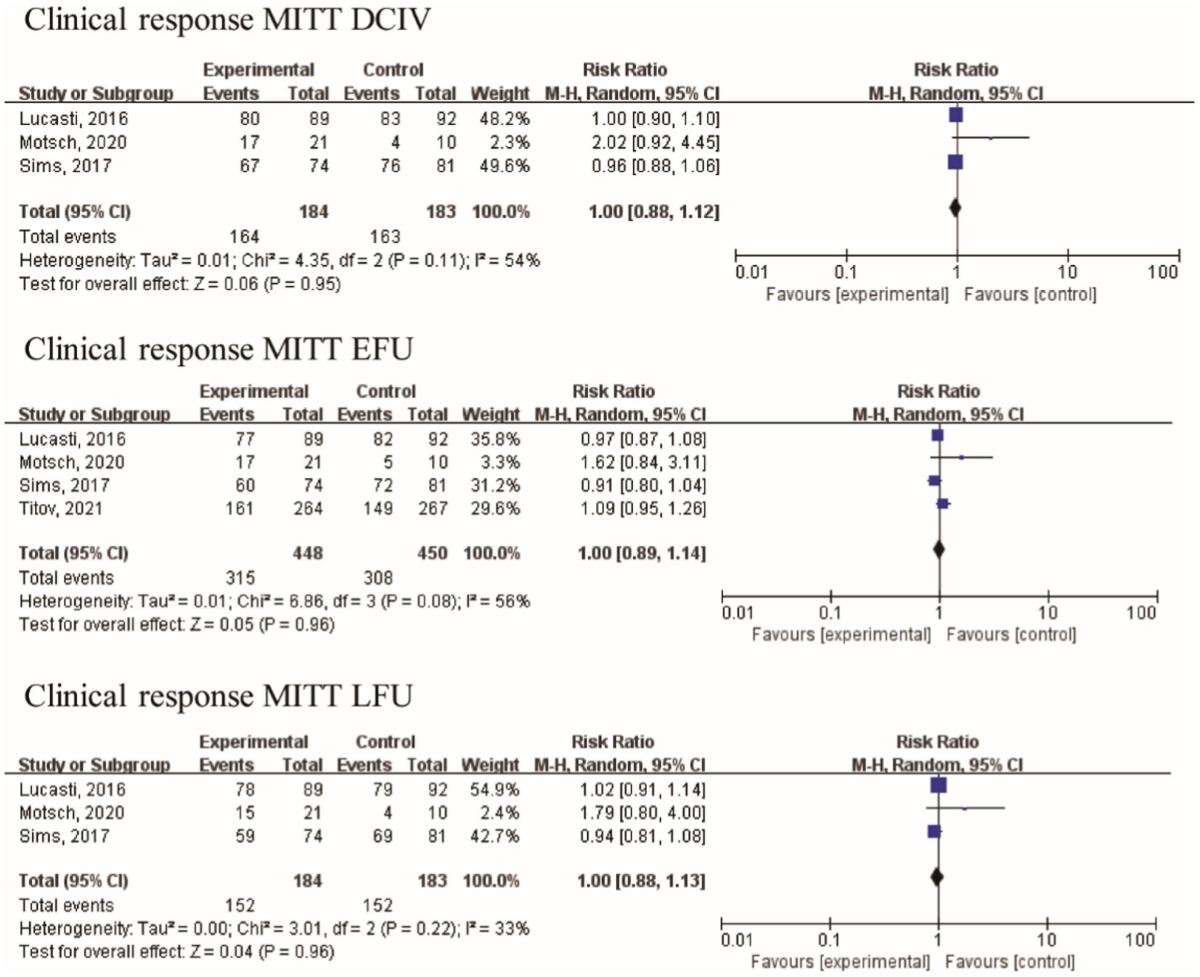
Overall clinical response in clinically relevant patient of the MITT population. MITT, microbiologic intent-to-treat; DCIV, discontinuation of intravenously therapy; EFU, early follow-up; LFU, late follow-up.

Efficacy in different populations was evaluated at three distinct visits: discontinuation of intravenously administered therapy (DCIV), which occurred between Day 5 and Day 14; early follow-up (EFU), which took place 5–9 days after the completion of all study therapy; and late follow-up (LFU), scheduled between 28–42 days after the completion of all study therapy (17, 18). In the pooled analysis of four studies, at the DCIV visit, the proportions of subjects in the microbiologic intent-to-treat (MITT) patients with a favorable clinical response were generally similar among the IMI/REL and comparator treatment groups [RR = 1.00 (0.88, 1.12), I^2^ = 54%, Figure 4]. And no statistically significant difference in the clinical response of MITT patients at the EFU visits [RR = 1.00 (0.89, 1.14), I^2^ = 56%] and at the LFU visits [RR = 1.00 (0.88, 1.13), I^2^ = 33%] was observed between IMI/REL and comparators.

**Figure 5:**
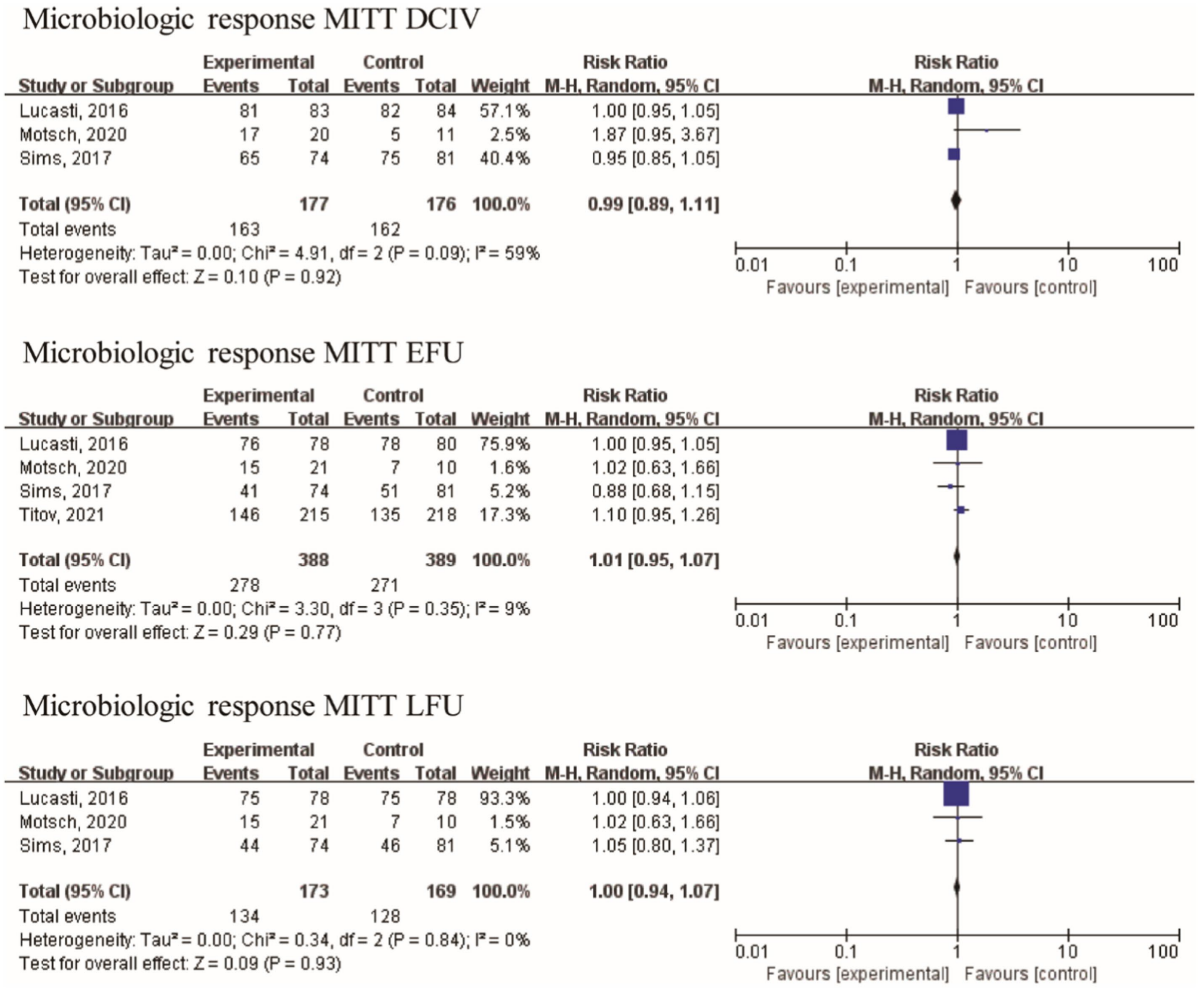
Overall microbiologic response in clinically relevant patient of the MITT population.

The baseline assessment revealed the presence of the following frequently encountered pathogens: *A. baumannii* (70 isolates), *E. coli* (266 isolates), *K. pneumoniae* (160 isolates), and *P. aeruginosa* (139 isolates). With the exception of Motsch’s study (17), the other three studies included a limited number of gram-positive bacteria in their analysis. *Bacteroides fragilis* and *Bacteroides thetaiotaomicron* were among the gram-negative anaerobic bacilli studied by Lucasti. Furthermore, the data related to the microbiological response were analyzed from four studies with 549 patients. No statistically significant difference in the microbiologic response of MITT patients at the DCIV visits [RR = 0.99 (0.89, 1.11), I^2^ = 59%, Figure 5], at the EFU visits [RR = 1.01 (0.95, 1.07), I^2^ = 9%], and at the LFU visits [RR = 1.00 (0.94, 1.07), I^2^ = 0%] was observed between IMI/REL and comparators in the pooled analysis.

### Safety

**Figure 6:**
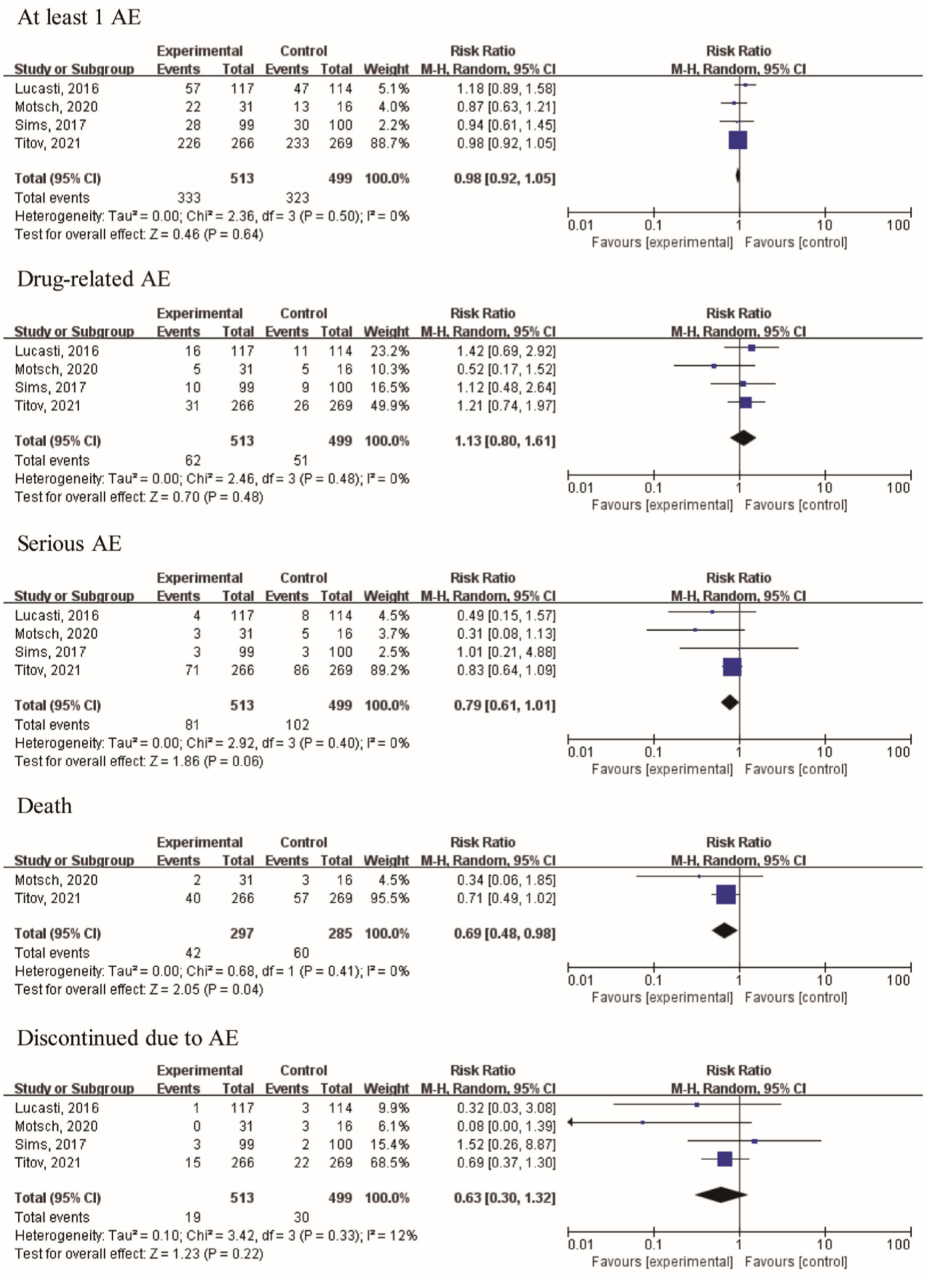
Risk of adverse events in clinically relevant patient.

Furthermore, two articles provided data on day-28 all-cause mortality and deaths. The risk ratios (RRs) of day 28 all-cause mortality and death were 0.71 [95% CI (0.49, 1.03), I^2^ = 1%, *p* = 0.07] and 0.69 [95% CI (0.48, 0.98); I^2^ = 0%, *p* = 0.04], respectively. The results indicated that treatment using the IMI/REL led to a statistically significant improvement in the survival rates. The therapy resulted in a similar risk of at least one AE [RR = 0.98 (0.92, 1.05), I^2^ = 0%, Figure 6], drug-related AEs [RR = 1.13 (0.80, 1.61), I^2^ = 0%], and discontinuation due to AEs [RR = 0.63 (0.30, 1.32), I^2^ = 12%] between the IMI/REL and comparators. And the serious AEs [SAEs, RR = 0.79 (0.61, 1.01), I^2^ = 0%] was significantly lower in the IMI/REL treatment group than in the comparator drug treatment group. Finally, the gastrointestinal disorders are the main AEs, but there was no significant difference between IMI/REL and comparators in the risk of gastrointestinal disorders, including diarrhea [RR = 1.20 (0.61, 2.35), I^2^ = 0%, Table 3], nausea [RR = 0.83 (0.42, 1.64), I^2^ = 0%], and vomiting [RR = 1.76 (0.61, 5.13), I^2^ = 0%].

**Table 3:** The results of gastrointestinal adverse events in meta-analysis.

Intervention	Relebactam (N/N)	Comparator (N/N)	*I*^2^	Effect estimate	*p* value
Diarrhea	18/482	15/483	0	1.20 [0.61, 2.35]	0.60
Nausea	15/513	16/499	0	0.83 [0.42, 1.64]	0.59
Vomiting	10/414	5/399	0	1.76 [0.61, 5.13]	0.30

## Discussion

Currently, carbapenem antibacterial drugs have been widely used for over 40 years in the treatment of infectious diseases. But because of the overuse and misuse of carbapenem antibacterial drugs, more carbapenem-resistant bacteria have been isolated from hospitals (19). Bacterial carbapenemase production and carbapenem response activity could be inhibited by -lactamase inhibitors. IMI/REL, a novel combination of -lactamase inhibitors and antibacterial agents, has been approved by the FDA for VABP, cUTIs, and cIAIs (4, 20). However, in recent years, the use of IMI/REL has expanded to include the treatment of other bacterial infections due to the emergence of drug-resistant bacteria. A meta-analysis of safety and efficacy data collected from four randomized controlled trials involving patients with bacterial infections found no statistically significant difference in clinical efficacy between IMI/REL and comparator treatments in adult patients.

The clinical and microbiological responses to IMI/REL in the treatment of bacterial infections were found to be comparable to the responses observed with the comparator therapies across all pooled populations included in the four randomized controlled trials (RCTs). For the complicated intra-abdominal infection, the results are consistent with the studies by Lucasti’s (16) and Sims’s (15). Similarly, a study by Yu’s (21) found that IMI/REL and meropenem-vaborbactam outperformed comparators in terms of clinical cure and survival rate in the treatment of complicated infections. But the results of a study by Motsch’s indicated that IMI/REL is an efficacious and well-tolerated treatment option for carbapenem-non-susceptible infections (17). These findings were supported by the results of the MIC test in a study by Hernández-Garca. According to the findings of this study, IMII and REL were active against 100% of ESBLs from *E. coli* and *Klebsiella* spp. isolates and 80.4% of carbapenemase from *Klebsiella* spp. producers (22). Furthermore, the results of Yang’s study (23) showed that IMI/REL significantly improved antibacterial activity with MIC_50_ (from 16 to 0.5 g/mL), MIC_90_ (from >32 to 4 g/mL), and susceptibility (from 18.9 to 82.2%). Like IMI and meropenem, IMI/REL is only available intravenously and has activity against gram-positive and gram-negative bacteria (19). Overall, IMI/REL provides a viable treatment option for patients with carbapenemase-producing bacterial infections such as ESBL, CRE, and KPC-positive infections. Second, both the IMI/REL and the comparators had a similar risk of at least one AE, drug-related AEs, and discontinuation due to AEs. SAEs were significantly lower in the IMI/REL than in the comparison groups. Gastrointestinal disorders are the main AEs, but there is no significant difference between IMI/REL and the comparators.

Another significant consideration regarding the treatment of bacterial infections with IMI or REL is the safety risk associated with AEs. All of the studies included in the analysis reported the occurrence of AEs in patients receiving IMI/REL and comparators, with varying frequencies and severities. In this analysis, the pooled risks of at least one AE, drug-related AEs, and discontinuation due to AEs were similar between IMI/REL and comparators. The risk of SAEs was significantly lower inIMI/REL than in the comparators. However, the overall incidence of SAEs was lower than that in Titov’s study (18), but higher than in the other three included studies (15–17). Moreover, the most common adverse events are nausea, diarrhea, and vomiting. The meta-analysis found that the risk of gastrointestinal disorders was comparable between IMII and REL, as well as among the comparators. However, in Motsch’s study, however, the risk of nausea in IMII and REL was significantly lower than that in the comparators (17). AEs such as decreased renal clearance of creatinine, hyperglycemia, infusion site erythema, pyrexia, dizziness, increased alanine aminotransferase, increased aspartate aminotransferase, increased blood creatinine, oral hypoesthesia, and leukopenia were also discovered in the studies. And the above-mentioned adverse reactions are still listed on the IMI/REL product labels based on clinical experience and safety data from clinical trials.

## Conclusion

In conclusion, the clinical response, microbiologic response, and risk of AEs of IMI/REL in the treatment of bacterial infections were similar to those of the comparator, and this antibiotic is as well tolerated as the comparator. Therefore, IMI/REL can be considered as an alternative treatment agent. The combination of IMI/REL has been shown to be an effective therapeutic strategy, leading to positive outcomes in treated patients. However, in order to further confirm the conclusions regarding the clinical use of this combination, the inclusion of big clinical data or a larger sample size of RCTs is urgently required. These measures would yield more robust evidence to support the validity of IMI/REL in the field of medicine.

## Data availability statement

The original contributions presented in the study are included in the article/supplementary material, further inquiries can be directed to the corresponding author.

## Author contributions

QY: Data curation, Investigation, Writing – original draft. YY: Data curation, Investigation, Writing – review & editing. RH: Formal analysis, Software, Writing – review & editing. BY: Data curation, Investigation, Writing – review & editing. YZ: Formal analysis, Software, Writing – review & editing. FL: Conceptualization, Methodology, Writing – review & editing.

## References

[1] Wang J Song Y Huang Z Lin W Yu G Xiong Y. Coupling a virulence-targeting moiety with Ru-based AMP mimics efficiently improved its anti-infective potency and therapeutic index. J Med Chem. (2023) 66:13304–18. doi: 10.1021/acs.jmedchem.3c01282, PMID: 37704628

[2] Doi Y. Treatment options for Carbapenem-resistant gram-negative bacterial infections. Clin Infect Dis. (2019) 69:S565–75. doi: 10.1093/cid/ciz830, PMID: 31724043 PMC6853760

[3] Al-Hasan MN. Gram-negative Bacteria with difficult-to-treat resistance: a moving target. Clin Infect Dis. (2021) 72:2121–3. doi: 10.1093/cid/ciaa384 32249916

[4] Sellares-Nadal J Eremiev S Burgos J Almirante B. An overview of cilastatin + imipenem + relebactam as a therapeutic option for hospital-acquired and ventilator-associated bacterial pneumonia: evidence to date. Expert Opin Pharmacother. (2021) 22:1521–31. doi: 10.1080/14656566.2021.1939680, PMID: 34120547

[5] Gaibani P Giani T Bovo F Lombardo D Amadesi S Lazzarotto T. Resistance to ceftazidime/avibactam, Meropenem/Vaborbactam and imipenem/Relebactam in gram-negative MDR Bacilli: molecular mechanisms and susceptibility testing. Antibiotics (Basel). (2022) 11:11(5). doi: 10.3390/antibiotics11050628 PMC9137602 35625273

[6] O'Donnell JN Lodise TP. New perspectives on antimicrobial agents: imipenem-Relebactam. Antimicrob Agents Chemother. (2022) 66:e0025622. doi: 10.1128/aac.00256-22, PMID: 35727059 PMC9295581

[7] Hujer AM Bethel CR Taracila MA Marshall SH Rojas LJ Winkler ML. Imipenem/Relebactam resistance in clinical isolates of extensively drug resistant *Pseudomonas aeruginosa*: inhibitor-resistant beta-lactamases and their increasing importance. Antimicrob Agents Chemother. (2022) 66:e0179021. doi: 10.1128/aac.01790-21, PMID: 35435707 PMC9112901

[8] Mansour H Ouweini AEL Chahine EB Karaoui LR. Imipenem/cilastatin/relebactam: a new carbapenem β-lactamase inhibitor combination. Am J Health-Syst Pharm. (2021) 78:674–83. doi: 10.1093/ajhp/zxab012, PMID: 33580649

[9] Castanheira M Doyle TB Deshpande LM Mendes RE Sader HS. Activity of ceftazidime/avibactam, meropenem/vaborbactam and imipenem/relebactam against carbapenemase-negative carbapenem-resistant Enterobacterales isolates from US hospitals. Int J Antimicrob Agents. (2021) 58:106439. doi: 10.1016/j.ijantimicag.2021.106439, PMID: 34547421

[10] Reviriego C. Relebactam. Beta-lactamase inhibitor Drugs of the Future. (2015) 40:0717. doi: 10.1358/dof.2015.040.11.2359467

[11] Moher D Shamseer L Clarke M Ghersi D Liberati A Petticrew M. Preferred reporting items for systematic review and meta-analysis protocols (PRISMA-P) 2015 statement. Syst Rev. (2015) 4:1. doi: 10.1186/2046-4053-4-1 25554246 PMC4320440

[12] Puljak L Ramic I Arriola Naharro C Brezova J Lin YC Surdila AA. Cochrane risk of bias tool was used inadequately in the majority of non-Cochrane systematic reviews. J Clin Epidemiol. (2020) 123:114–9. doi: 10.1016/j.jclinepi.2020.03.019, PMID: 32247026

[13] Higgins JPT G.S., Cochrane handbook for systematic reviews of interventions version 5.1.0, (2011). Available at: http://handbook-5-1.cochrane.org/

[14] Guyatt GH Oxman AD Vist GE Kunz R Falck-Ytter Y Alonso-Coello P. GRADE: an emerging consensus on rating quality of evidence and strength of recommendations. BMJ. (2008) 336:924–6. doi: 10.1136/bmj.39489.470347.AD, PMID: 18436948 PMC2335261

[15] Sims M Mariyanovski V McLeroth P Akers W Lee YC Brown ML. Prospective, randomized, double-blind, phase 2 dose-ranging study comparing efficacy and safety of imipenem/cilastatin plus relebactam with imipenem/cilastatin alone in patients with complicated urinary tract infections. J Antimicrob Chemother. (2017) 72:2616–26. doi: 10.1093/jac/dkx139, PMID: 28575389

[16] Lucasti C Vasile L Sandesc D Venskutonis D McLeroth P Lala M. Phase 2, dose-ranging study of Relebactam with imipenem-Cilastatin in subjects with complicated intra-abdominal infection. Antimicrob Agents Chemother. (2016) 60:6234–43. doi: 10.1128/AAC.00633-16, PMID: 27503659 PMC5038313

[17] Motsch J de Oliveira CM Stus V Koksal I Lyulko O Boucher HW. RESTORE-IMI 1: a multicenter, randomized, double-blind trial comparing efficacy and safety of imipenem/Relebactam vs Colistin plus imipenem in patients with imipenem-nonsusceptible bacterial infections. Clin Infect Dis. (2020) 70:1799–808. doi: 10.1093/cid/ciz530 31400759 PMC7156774

[18] Wunderink TIRG Roquilly A Gonzalez DR David-Wang A Boucher HW Kaye KS. Randomized, double-blind, multicenter trial comparing efficacy and safety of imipenem/Cilastatin/Relebactam versus piperacillin/Tazobactam in adults with hospital-acquired or ventilator-associated bacterial pneumonia (RESTORE-IMI 2 study). Clin Infect Dis. (2021) 73:e4539–48. doi: 10.1093/cid/ciaa803 32785589 PMC8662781

[19] Zhanel GG Lawrence CK Adam H Schweizer F Zelenitsky S Zhanel M. Imipenem-Relebactam and Meropenem-Vaborbactam: two novel Carbapenem-beta-lactamase inhibitor combinations. Drugs. (2018) 78:65–98. doi: 10.1007/s40265-017-0851-9, PMID: 29230684

[20] Luci G Mattioli F Falcone M Di Paolo A. Pharmacokinetics of non-beta-lactam beta-lactamase inhibitors. Antibiotics (Basel). (2021) 10:7. doi: 10.3390/antibiotics10070769 PMC8300739 34202609

[21] Yu W Shen P Luo Q Xiong L Xiao Y. Efficacy and safety of novel carbapenem-beta-lactamase inhibitor combinations: results from phase II and III trials. Front Cell Infect Microbiol. (2022) 12:925662. doi: 10.3389/fcimb.2022.925662, PMID: 36211957 PMC9538188

[22] Hernandez-Garcia M Garcia-Castillo M Bou G Cercenado E Delgado-Valverde M Oliver A. Imipenem-Relebactam susceptibility in Enterobacterales isolates recovered from ICU patients from Spain and Portugal (SUPERIOR and STEP studies). Microbiol Spectr. (2022) 10:e0292722. doi: 10.1128/spectrum.02927-22, PMID: 36043877 PMC9602286

[23] Yang TY Hsieh YJ Kao LT Liu GH Lian SH Wang LC. Activities of imipenem-relebactam combination against carbapenem-nonsusceptible Enterobacteriaceae in Taiwan. J Microbiol Immunol Infect. (2022) 55:86–94. doi: 10.1016/j.jmii.2021.02.001, PMID: 33678555

